# Supplementary material for: Engineered natural killer cells impede the immunometabolic CD73-adenosine axis in solid tumors
Source: eLife. 2022 Jul 11;11:e73699. doi: 10.7554/eLife.73699 (PMC9342955; doi:10.7554/eLife.73699)
Supplement: Figure 1—source data 1. [file elife-73699-fig1-data1.docx]

**Figure 1 – Source Data 1**. **Raw NES and pvalues for TCGA analyses.** Raw NES and pvalues for significant pathways (pvalue < 0.25) related to natural killer cells in TCGA-LUAD patients between tumor and normal samples.

| ***NAME*** | ***NES*** | ***Pvalue*** |
| --- | --- | --- |
| GO POSITIVE REGULATION OF NK CELL DIFFERENTIATION | -1.502616604 | 0.073800738 |
| GO POSITIVE REGULATION OF NK CELL MEDIATED CYTOTOXICITY | -1.320404853 | 0.130952381 |
| GO POSITIVE REGULATION OF NK CELL ACTIVATION | -1.315517755 | 0.16091954 |
| GO POSITIVE REGULATION OF NK CELL MEDIATED IMMUNE RESPONSE TO TUMOR CELL | -1.28578655 | 0.178694158 |
| KEGG NK CELL MEDIATED CYTOTOXICITY | -1.214399204 | 0.142857143 |
| GO POSITIVE REGULATION OF NK CELL MEDIATED IMMUNITY | -1.191704891 | 0.233576642 |
| GSE37301 COMMON LYMPHOID PROGENITOR VS RAG2 KO NK CELL UP | 1.128881743 | 0.203 |
| GSE37301 LYMPHOID PRIMED MPP VS RAG2 KO NK CELL DN | 1.233385767 | 0.065 |
| GO POSITIVE REGULATION OF NK CELL CHEMOTAXIS | 1.273694139 | 0.156432749 |
| GO REGULATION OF NK CELL DIFFERENTIATION | 1.288360057 | 0.163913596 |
| GSE7764 IL15 TREATED VS CTRL NK CELL 24H UP | 1.316525898 | 0.011 |
| GO REGULATION OF NK CELL DIFFERENTIATION INVOLVED IN IMMUNE RESPONSE | 1.378315357 | 0.063694268 |
| GSE45365 NK CELL VS CD11B DC UP | 1.400662545 | 0.001 |
| GO NK CELL DIFFERENTIATION | 1.408656735 | 0.061151079 |
| GSE39556 CD8A DC VS NK CELL MOUSE 3H POST POLYIC INJ UP | 1.576097321 | 0.001 |
