# Supplementary material for: Engineered natural killer cells impede the immunometabolic CD73-adenosine axis in solid tumors
Source: eLife. 2022 Jul 11;11:e73699. doi: 10.7554/eLife.73699 (PMC9342955; doi:10.7554/eLife.73699)
Supplement: Figure 1—source data 2. [file elife-73699-fig1-data2.docx]

**Figure 1 – Source Data 2.** **Raw NES and pvalues for significant pathways stratified for *NT5E***. Raw NES and pvalues for significant pathways (pvalue < 0.25) related to natural killer cells in TCGA-LUAD patients between high and low groups stratified by *NT5E* gene expression.

| ***NAME*** | ***NES*** | ***Pvalue*** |
| --- | --- | --- |
| GO NK CELL ACTIVATION | 1.461626 | 0.023622 |
| GO NEGATIVE REGULATION OF NK CELL MEDIATED IMMUNITY | 1.566925 | 0.073684 |
| GO NK CELL CHEMOTAXIS | 1.524309 | 0.095395 |
| GO REGULATION OF NK CELL CHEMOTAXIS | 1.483928 | 0.116766 |
| GO NK CELL DEGRANULATION | 1.350595 | 0.182635 |
| GO ACTIN CROSSLINK FORMATION | 1.435619 | 0.126183 |
| GSE45365 NK CELL VS CD11B DC DN | 1.715205 | 0.022222 |
| GSE45365 NK CELL VS CD11B DC UP | -1.58869 | 0.001048 |
| GSE21774 CD62L POS CD56 BRIGHT VS CD62L NEG CD56 DIM NK CELL UP | -1.15955 | 0.194589 |
| GSE7764 IL15 TREATED VS CTRL NK CELL 24H UP | -1.14181 | 0.225941 |
